# The Impact of Road Construction on Subjective Well-Being in Communities in Madre de Dios, Peru

**DOI:** 10.3390/ijerph15061271

**Published:** 2018-06-15

**Authors:** Amy R. Riley-Powell, Gwenyth O. Lee, Nehal S. Naik, Kelly E. Jensen, Christina O’Neal, Gabriela Salmón-Mulanovich, Stella M. Hartinger, Daniel G. Bausch, Valerie A. Paz-Soldan

**Affiliations:** 1 Tulane University School of Public Health and Tropical Medicine, 1440 Canal St, New Orleans, LA 70112, USA; amyrileypowell@gmail.com (A.R.R.-P.); gwenyth.lee@gmail.com (G.O.L.); kjensen1@tulane.edu (K.E.J.); coneal3@tulane.edu (C.O.); Daniel.Bausch@phe.gov.uk (D.G.B.); 2 School of Public Health, University of Michigan, 1415 Washington Heights, Ann Arbor, MI 48109, USA; 3 School of Medicine, Virginia Commonwealth University, 1201 E Marshall St, Richmond, VA 23298, USA; nnaik27@gmail.com; 4 Tulane University School of Medicine, 1430 Tulane Ave, New Orleans, LA 70112, USA; 5 U.S. Naval Medical Research Unit No. 6, Callao, Callao 2, Peru; gsalmonm.veid@gmail.com; 6 School of Public Health and Administration, Universidad Peruana Cayetano Heredia, Ave Honorio Delgado 430, San Martín de Porres, Lima 31, Peru; stella.hartinger.p@upch.pe; 7 Biomedical Engineering, Pontificia Universidad Católica del Perú, Av. Universitaria 1801, San Miguel, Lima 32, Peru; 8 Swiss Tropical and Public Health Institute, Socinstrasse 57, 4051 Basel, Switzerland; 9 Swiss Tropical and Public Health Institute, University of Basel, Petersplatz 1, 4001 Basel, Switzerland

**Keywords:** well-being, Peru, environment, Latin America, mixed methodology

## Abstract

The interoceanic highway (IOH) in Madre de Dios, Peru has driven dramatic change in the Peruvian Amazon basin. We conducted a mixed methods study to examine the impact of these changes on the subjective well-being (SWB) of four communities on the IOH. Themes that emerged qualitatively included changing health threats, environmental degradation, and the impact of increased migration. To achieve a higher level of SWB, respondents emphasized the need for higher incomes, opportunities to learn new skills, and a better education for their children. Potential threats to SWB included marital problems and poorer health. Quantitative analyses suggested that social support and a sense of security impacted reported SWB scores based on life satisfaction, and the impact of income on life satisfaction was mediated by food security. Although long-term residents felt that specific determinants of SWB had both increased (food variety, transport and access to work) and decreased (access to natural resources and hunting), the majority reported that their lives had improved overall. Health had been affected by the IOH in both negative ways (increased dengue and road accidents) and positive ways (improved access to health services). Our results suggest that the rapidly-changing communities near the IOH link well-being to health, income, community, and the environment.

## 1. Introduction

New roads have important implications for the previously isolated communities affected by their construction. In the Amazon basin, the deforestation and land degradation often associated with these projects can dramatically change relationships between people and their environment. This may include limiting rural livelihood options or generating new economic opportunities, and increasing access to infrastructure or putting stress on existing services [1]. As a result, the impact of these projects on well-being and poverty is complex, and relatively little is known about how communities perceive these changes.

Well-being is a measure of human quality of life, inextricably linked to socio-ecological systems [2,3]. Subjective well-being (SWB) refers to a person’s self-reported well-being. Although eudaimonic definitions of well-being also exist, SWB is most often defined based on perceptions of life satisfaction or life evaluation and by the presence of positive mood and absence of negative mood (emotional or hedonic well-being) [4]. In contrast, objective well-being focuses on indicators of success such as life expectancy and income [5,6,7]. The relationship between indicators of objective well-being and SWB is an ongoing area of research, but determinants of SWB often include both personal characteristics (e.g., age, health, income) and external factors (e.g., governance, values, or religion). However, there is debate as to the relative importance or causal relationships between these factors [8]. This is particularly true in low and middle-income countries, where cultural differences in the components of SWB may exist, and among rural households that depend more directly on the natural environment for food and income. In this context, variability or disruptions to socio-ecological systems can have a substantial direct impact on SWB [3,9]. The ‘buen vivir’ (living well) social movement, which rose to prominence in Latin America in the 2000s, emphasizes the interconnectedness of the individual, the community, and the natural environment, and the effect of this nexus on SWB [10]. A related concept is ‘allin kawsay’ which has its historical origins in Andean conceptions of well-being and emphasizes the interdependence of these factors and specifically how they relate to safeguarding food sovereignty and food security [11]. There is a need to better understand how these local definitions and determinants of SWB are impacted by structural and environmental change. In particular, studies in the Amazonian and Andean regions have found higher levels of SWB in more isolated communities, which decreased with modernization [12]. Studies in high-income countries have also found that new roads can decrease physical and mental wellbeing, and negatively impact health [13].

The interoceanic highway (IOH) joins the Pacific Ocean in Peru with the Atlantic Ocean in Brazil. Paved in 2006, the highway traverses the Peruvian Amazonian region of Madre de Dios and goes through the regional capital, Puerto Maldonado (pop. 109,555 [14]). Madre de Dios is characterized as a rainforest ecosystem with tremendous biodiversity [15,16]. The IOH has been a driver of change—with increased movement of people and goods, and changes to economic opportunities, as well as illegal gold mining and logging [15,17,18]—which has contributed to a decrease in biodiversity of flora and fauna of the region. Migration has rapidly increased, leading to the growth of new settlements and changes to cultural and societal norms within communities [19]. At the same time, changes in land use have been associated with an increased incidence of malaria [18], metal poisoning, and possible increases in rodent borne disease [15,18,20]. However, the IOH has also been associated with improved health through better health system accessibility. With these changes also come potential effects to the SWB of the communities, which is less documented.

We examined changing barriers and enablers of SWB, both qualitatively and quantitatively, in eight communities in Madre de Dios alongside the IOH. In the quantitative analysis, we defined SWB based on life satisfaction using a validated instrument—Happy Ladders [21]—to (1a) estimate SWB scores, both overall and disaggregated by migration history, gender, and age; (1b) examine household-level factors associated to SWB scores; and (2) described factors that community members reported as relating to their potential future well-being. In the qualitative exploration, we examined community perceptions of well-being and of how the IOH had affected well-being.

## 2. Materials and Methods

We conducted a mixed-methods study with an iterative process of qualitative and survey data collection and analysis to explore individual perceptions of well-being, as well as related topics including health, environmental change, and community dynamics. The study comprised (i) key informant interviews; (ii) focus group discussions with community members; and (iii) quantitative surveys. The project was embedded within a larger multi-disciplinary study examining anthropogenic change along the IOH and related changes in rodent population dynamics and risks of rodent-borne disease [15,19,22].

### 2.1. Development of Data Collection

The first phase of research consisted of qualitative focus group discussions (FGDs), and key informant interviews (KIIs), conducted in both 2014 and 2015; in 2014, the research took place in eight communities and in 2015 in four communities along the IOH. On both occasions, half the sampled communities were north and half were south of Puerto Maldonado. A primary objective of this qualitative work was to identify and explore perceptions of SWB and to ensure that individuals had an opportunity to identify the range of aspects of well-being that were important to them, thus helping the research team define what topics related to their SWB were of priority to them and should be included in the survey. Following analysis of this data (described in detail below), a quantitative survey was developed to examine identified themes as well as a priori topics of interest. This survey was organized according to an adapted version of the ‘Five Capitals of Sustainable Development—a theoretical framework widely used in vulnerability literature [23,24,25], This theoretical framework organizes rural household livelihood capacities as human (e.g., access to education, health); physical (land and water endowments); social (connectivity in social and political networks), financial (assets, monetary savings, income) and personal (household safety and security) [26]. Note that in the results section, we will refer only to elements within each of these five capitals that we identified to be most associated to well-being. For the second phase of data collection, the survey was administered to all occupied households in four communities (N = 522), and additional KIIs were conducted to examine the same issues from the perspective of local authorities.

### 2.2. Qualitative Methods

FGDs and KIIs were conducted by a trained Peruvian anthropologist (in 2014) and a trained researcher (in 2015) using semi-structured guides developed by the research team. Themes of interest included well-being, health, education, safety, as well as how each of these relate to the IOH. KIIs were focused on exploring community characteristics. Topics included history of the community, availability of individual and organizational resources, population characteristics, and any observed changes over the previous 5–10 years with the IOH construction being mentioned to prompt memories of changes.

Purposive sampling was used to recruit FGD participants. One to two days prior to the FGD, our team recruited participants from public areas, such as shops and restaurants. Due to low attendance rates, some participants were also recruited on the same day, using the same methods. In all but one instance, FGDs were stratified by gender, based on previous experience with FGDs in Peru revealing that single sex focus groups ensure more participation from all. Purposive and snowball sampling was also used to identify and recruit KII participants until saturation of topics was reached. In each community, we started by interviewing the community leader and/or community health professional and asked for introductions to other key informants in the community.

At least one note taker was present at each FGD and KII. Following each FGD or KII, the facilitator and note taker(s) compiled detailed summaries of the events. Interviews were audio-recorded when the key informant gave informed consent. Focus groups were not audio-recorded due to difficulty with the sound quality and detailed notes were used in lieu of transcriptions.

### 2.3. Survey

Every household within the four selected communities was invited to participate in the survey, which was administered by field workers trained by very experienced personnel overseeing the survey. Households were identified based on updated maps obtained from the local authorities and health care personnel of the communities. Within the survey, SWB was measured using the Happy Ladder instrument, a validated life satisfaction measure previously used in Peru [21]. Participants self-assessed their current well-being on a scale from zero to ten, where zero represented the worst possible place in the scale and ten the best. They were then prompted to describe threats or factors that might negatively impact their well-being and opportunities to improve their well-being [27]. The survey also included demographic information, and detailed questions about health, community participation, livelihoods, and assets.

### 2.4. Ethical Review and Informed Consent

This study protocol was approved by the institutional review boards of the Tulane University School of Public Health and Tropical Medicine in New Orleans (LA, USA, #545719), the Universidad Peruana Cayetano Heredia (Lima, Peru, #62162), and the US Naval Medical Research Unit No. 6 in Lima, Peru (#NMRCD.2013.0025). Prior to starting the study, community leaders were contacted and agreed to support the study, but requested that illegal gold mining, which is common in this region [18,28], not be discussed. Some key informants also declined to participate due to anxiety around this issue. For these reasons, despite the significant impact of illegal gold mining on many aspects of individual and community well-being, we did not ask questions about gold mining during the study. Written informed consent was obtained from all participants.

### 2.5. Qualitative Analysis

Qualitative data recordings were transcribed, and transcriptions and notes were uploaded to Dedoose, a web application for managing, analyzing, and presenting qualitative and mixed method research data [29]. A codebook was developed by the research team, focusing on themes identified during the data collection process. The codes were applied, changes were made based on additional themes that arose during the coding, and data were iteratively re-coded. Every fifth transcript was double coded to identify potential discrepancies which were then discussed and resolved between coders. This iterative is based on grounded theory and permits unbiased development of the results and key themes [30].

### 2.6. Quantitative Analysis

Survey data was used to characterize and quantify the prevalence of factors related to SWB, initially identified through KIIs and FGDs. Wealth was characterized using a validated index based on the presence of household utilities (electricity, water, gas, etc.), household construction materials, and ownership of domestic and electronic household goods. These were used to create a decimal score between 0 and 1 [31]. Improved water sources were defined based on the United Nations-World Health Organization Joint Monitoring Program standard definitions [32].

Data were described by mean and standard deviation for normally distributed data, median and interquartile range for non-normally distributed data, and frequencies for binary and categorical data. Spider plots were used to visually examine the prevalence of these variables by community membership, age, sex, and self-identified migrant status, defined in the survey as having moved to the community after paving of the IOH, or in 2006 or after [19]. ANOVA and Pearson’s chi square tests were used to test whether differences in the prevalence of these variables across these sub-groups were statistically significant. The frequency with which specific themes were mentioned in response to potential future improvements or decreases in SWB was also compared between groups. Bivariate linear regression models were then built to examine association between variables of interest and the Happy Ladder score. A multivariate model was developed to relate the same factors to SWB while controlling for age, sex, wealth index, and migrant status, with final variable selection informed by a priori study questions of interest and model fit.

For all analyses, statistical significance was set at a *p*-value of < 0.05, and survey data was analysed using Stata 14.1 (StataCorp, College Station, TX, USA) [33].

## 3. Results

### 3.1. Socio-Demographic Characteristics of Our Sample

#### 3.1.1. FGDs and KIIs

Twelve FGDs were conducted in 2014 with a total of 50 female and 33 male community members with no more than 13 participants per group, lasting ninety minutes on average. Twenty-one KIIs were held in 2014, and 13 were held in 2015. These included 17 KIIs with elected authorities, 10 with health professionals, five with education professionals, and two with community members with leadership positions (e.g., leader of community kitchen). A minimum of two and a maximum of seven interviews were conducted in each of the eight communities (Table 1).

#### 3.1.2. Surveys

Forty-one percent (41%) of homes were unoccupied at the time of the survey, likely reflecting the highly mobile nature of the population. Among occupied homes, the response rate was high, with only 6% of occupied households declining to participate and 8% unable to participate at the time of data collection [19]. A total of 522 surveys were administered. Survey data demographics are summarized in Table 2.

### 3.2. Findings

The results are presented based on the three aims of this manuscript. Rather than dividing the data presentation by type of data, findings from the qualitative (FGDs and KIIs) and quantitative (surveys) components are presented together based on the themes being addressed.

#### 3.2.1. (1a) Characterization of SWB and Its Determinants, Disaggregated by Community, Gender, Age, and Migration History

The SWB scoring is from 0 to 10, with 10 representing the best possible state of SWB, as based on the Happy Ladder instrument. The average SWB score across all participants was 4.4 (SD = 2.1). When asked what they thought their position on the Happy Ladder would be in four years’ time, the average level was 5.5. When asked what factors would help them to achieve a greater level of well-being, the most common responses were more money (71.3%), followed by learning new skills (24.5%), and better education for their children (12.3%). When asked what factors would adversely affect well-being, most common responses were marital/relationship problems (88.3%), poorer health (49.8%), and reduced income (39.1%).

**Figure 1:**
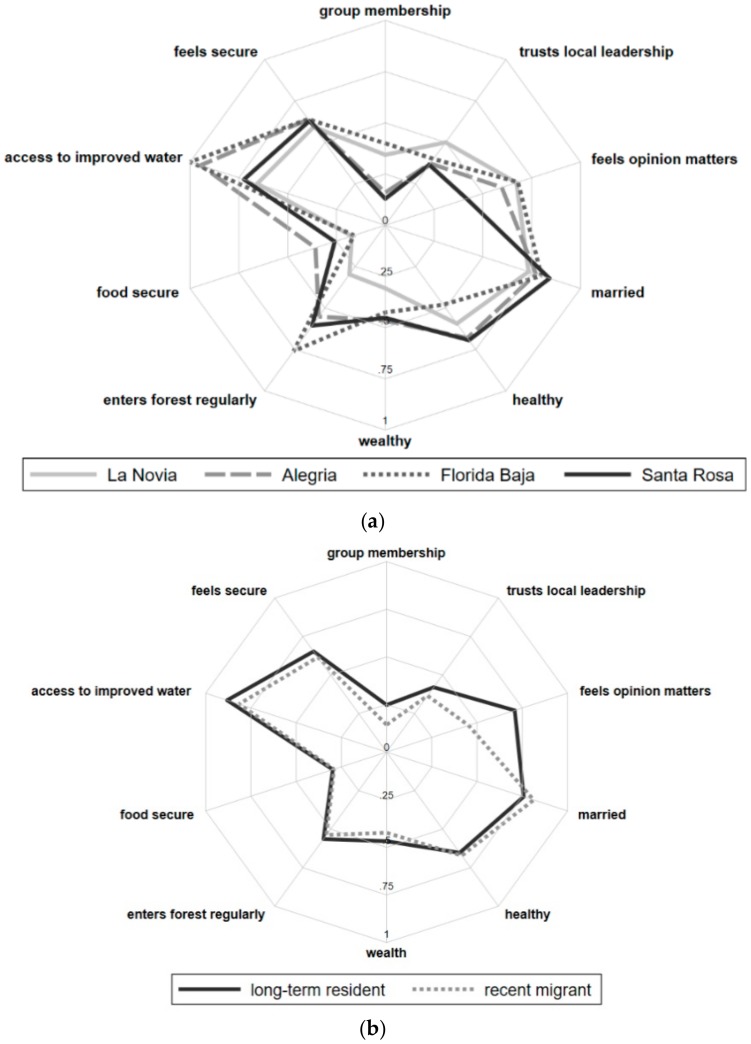

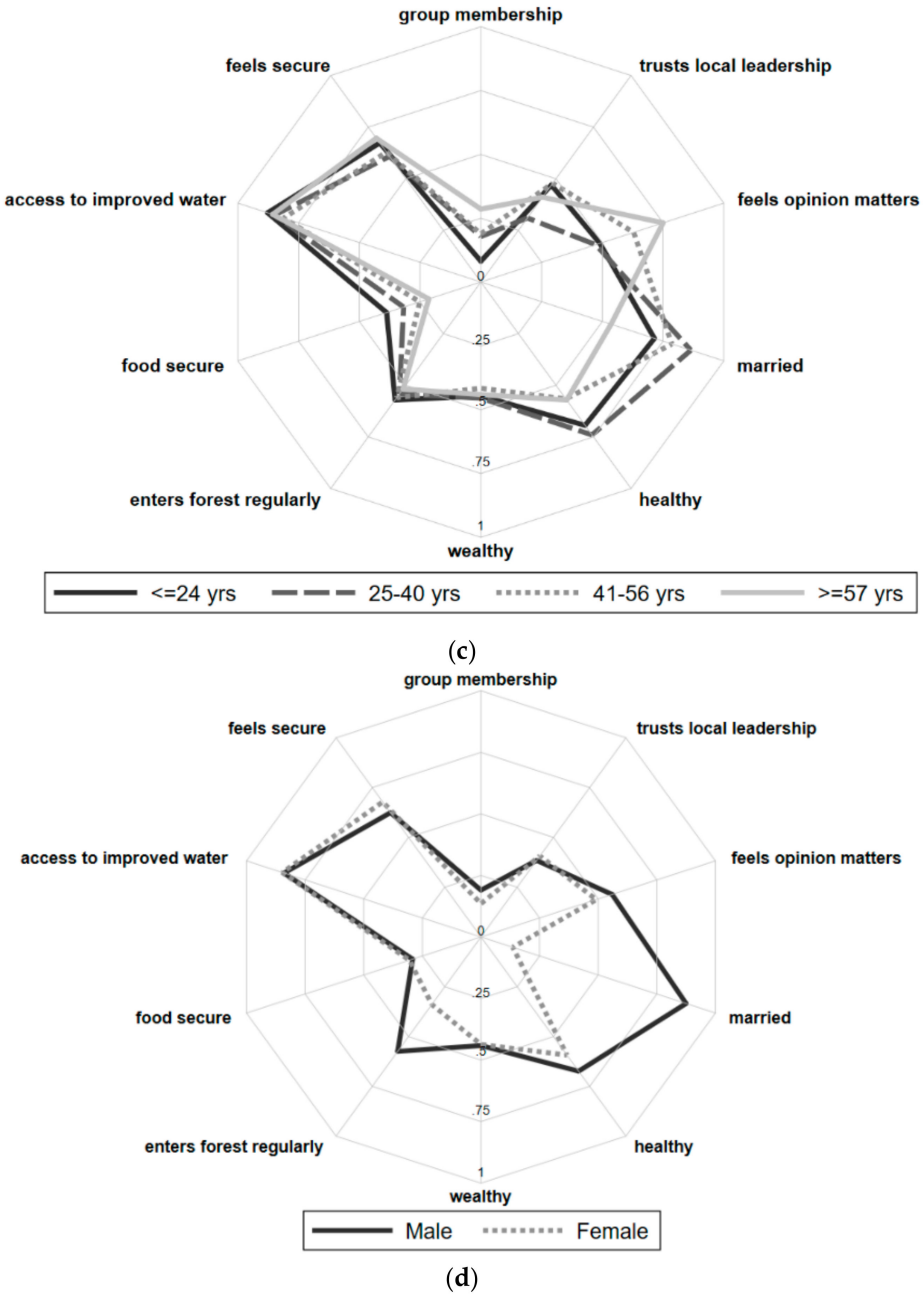
Spider plots of key variables by community. (**a**) key factors by community; (**b**) key factors by migration status (years in community); (**c**) key factors by age of head of household; (**d**) key factors by sex of head of household.

There were no significant differences in SWB Happy Ladder scores among the four communities (*p* = 0.315), and no differences in people’s sense of security or reported presence of health problems in the past year. There were differences in community social group membership, with more community group participation in Florida Baja and La Novia, (*p* < 0.001, χ^2^ test), more food security in Alegria (*p* < 0.001, χ^2^ test), greater access to improved water sources in Alegria and Florida Baja (*p* < 0.001, χ^2^ test), and lower wealth scores in La Novia (*p* < 0.001, χ^2^ test). (Figure 1).

Female-headed households reported marginally lower SWB Happy Ladder scores (*p* = 0.057, *t*-test) as compared to male-headed households. However, female- and male-headed households reported a similar sense of security, prevalence of health problems in past year, community group membership, access to improved drinking water, food security, and wealth. The age of the head of the household was not associated with SWB, sense of security, access to improved water, or wealth. Households with an older head reported more health problems in the past year (*p* = 0.001, *t*-test). Households with an older head were also marginally more likely to participate in community groups, but were marginally less food secure (*p* = 0.118, *t*-test). Sixty-one (61.3%) of survey respondents were migrants, of these 64% reported that their primary reason for moving to their community was economic [19]. Migrant households were less likely to report participation in community groups (*p* = 0.001, χ^2^ test), and had lower wealth scores (0.001, *t*-test). However, migrant households did not report differences in their sense of security, presence of health problems in the past year, access to improved water, or food security.

#### 3.2.2. (1b) Factors Associated with SWB Scores

We examined variables that might be associated with higher SWB scores using bivariate regression modelling, and found that these included a feeling of security, participation in community groups, and food security. Individuals living in larger households also reported greater SWB. Greater wealth and access to an improved water source were associated with SWB in bi-variable but not multi-variable regression models, suggesting that the role of these factors on SWB was mediated by other factors. Specifically, after adjusting for food security, the relationship between wealth and SWB was attenuated (Table 3).

#### 3.2.3. (2) Perceptions of SWB and of How the IOH Had Affected SWB.

**Figure 2:**
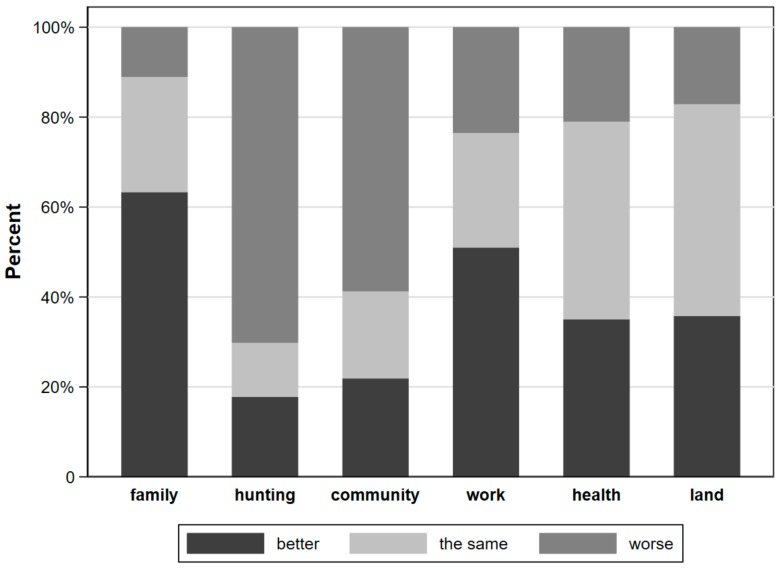
Perception of changes in each of the five capitals (is X is better/worse/the same since the IOH), were asked of long-term residents. Examples of questions relating to capitals include, Physical = access to hunting, fishing and forest products; Social = your community; Financial = possibilities for work; Human = your health; Personal = your family.

There were both similarities and differences in the themes that emerged in the qualitative and quantitative results. Based on data from the surveys, since the IOH construction, 63.3% of long-term residents felt that their lives had improved, 25.6% of long-term residents felt that life was the same, and only 11.1% felt that it had gotten worse (Figure 2). The major themes that came up during the qualitative FGDs and KIIs regarding the impact of the IOH on their well-being related to changes in specific components of social capital (specifically, migration), human capital (i.e., health), and physical capital (i.e., environmental change). These are described below along with quotations to support our findings.

### 3.3. Wealth

Wealth was raised in the qualitative portion of the study but often through concerns about land ownership and limited access to loans or credit. In the survey, most households (71.2%) described ‘more money’ as an option for increasing position on the Happy Ladder, but only 5.8% (30/522) specifically mentioned access to government loans. A key informant (KI) working in education reported the “*main problem that they have is the [official] possession of land*” (0102). This was often mentioned alongside feelings of frustration regarding ‘official’ administration and feeling forgotten by the government. Key informants expressed concerns such as, “[I don’t like] *that we are forgotten by the government*” (0403) and “*there should be more state presence here*” (0103). There was also a distrust of the role of government and government bodies in preventing illegal activity; “*there is no support from the state, the state simply gives [the neighborhood watch] a vest but you cannot do anything to a criminal, you cannot. On the contrary, the criminal can kill you. Even the authorities are threatened.*” (0202).

### 3.4. Migration

During KIIs, concerns were expressed about the growing migrant community. Perceptions of migrants were primarily negative, as migrants were considered to have brought about increased delinquency, including drug/alcohol abuse, and theft to the communities. One KI who was an elected authority described his dislike:

“I do not like that there are too many parties that they [the migrants] come from all places, to the road they come. I do not know what kind of people they will be? Maybe the town is attacked, because we are peaceful people, so when a group of 10–15 thugs come who are drugged and do things… How has this happened in other towns? This happens in other towns, in Ayacucho [Andean state, recognized as a location where terrorism grew], in these types of places they destroy the community and this is why sometimes you do not sleep. It’s worse that we are on the road and we have no security, so where do we go?”

(0304)

A healthcare worker also described the difficulty they face with patient follow-up care as it pertains to migrants, saying, “What is difficult for us is the presence of migrants and patient follow-up. Because when we go to see them in their houses, they have returned to their towns and we can’t complete their care. It’s something that goes against how we work.” (0203). On the other hand, migrants were also considered by some community members to have brought about, “increased amounts of cultural exchange... [the IOH] has improved the quality of life.” (0401). This sentiment was heard a few times in the KIIs and FGDs as a positive outcome of the IOH.

### 3.5. Health

Whilst health issues were not associated with SWB scores in survey data, the theme of health and access to health services was featured prominently in perceptions of SWB. Health issues included dengue fever, which 6.7% (N = 35) of households reported having experienced in the past year, making it the most common specific health problem described, and road traffic accidents, which 2.1% (N = 11) of households reported. Most respondents felt that factors related to health were similar (44.0%) or better (35.0%) to what it had been before the IOH (compared to 21.0% who felt it was worse), and that factors related to work opportunities were better (51.0%) or similar (25.5%), compared to worse (23.5%), since the IOH. Among threats to health, the burden from infectious diseases was most often raised. Dengue was also raised in all FGDs and in some KIIs, with participants emphasizing its high prevalence and associated mortality. One KI stated: “*There are a lot of cases, hemorrhagic dengue has killed people on a few occasions; everyone has had dengue, there isn’t anyone that hasn’t had it*” (0201). Community members reported sparse health campaigns to address both dengue and other diseases. However when health campaigns had happened, they were reportedly highly successful. In Santa Rosa, a KI described a vector control campaign that happens in suspected cases of dengue, “*Yesterday we were fumigating all day to eliminate the mosquitoes because we have had cases of dengue, suspected dengue, and we had to do emergency fumigation*” (0401). Various other infectious diseases were also brought up in both the focus groups and interviews, but less discussed, including diarrhea, colitis, leishmaniosis, fungal infections, and tuberculosis. The other threat to the health of the communities that was a direct result of the IOH were road traffic accidents, with one KI describing, “*the only defect that the IOH has brought is traffic accidents, there’s a lot of traffic accidents, deaths, almost weekly we have traffic accidents*” (0201).

Access to healthcare services was another prominent theme that arose during the qualitative data collection. The IOH was seen as having had a positive influence on access to healthcare services, by making it faster and easier to travel to the regional hospital in Puerto Maldonado if needed. In one FGD, community members mentioned that before the IOH, people would die if they were sick, because travel to Puerto Maldonado took three days. However, this relative proximity to better healthcare services did not appear to translate into better support from national and regional health services. The lack of government programs, support, and infrastructure was highlighted as a potential threat to health. One of the programs discussed includes the Health Service for Marginalized Rural and Urban communities (SERUM) program that places newly graduated doctors in rural, underserved areas to work for one year [34]. A KI commented on this program saying, “*Here there is not a [permanent] doctor, they only come with the program... a doctor and a nurse. They are assigned and rotate every year. It’s not guaranteed and every time they rotate they aren’t the same type of person*” (0304). In another community, where there is no health center, a KI reported that community members have to go to Puerto Maldonado for health care and that, “*we are fighting [for a health post] because there is a large enough population here*” (0103). This frustration was echoed by health professionals who reported not conducting “*health campaigns because of the small number of healthcare personnel so we cannot go out* [into the community]” (0401) and the apparent disinterest in primary healthcare services, “*They* [community members] *are mainly interested in showing off, having a house, a car, motorcycle, clothes, but health is the last thing*” (0401).

### 3.6. Environmental Change

Most survey respondents felt that access to natural resources such as wildlife, fishing, wood, and other forest products (factors associated to physical capital) had diminished since the construction of the IOH. Approximately seventy percent (70.2%) felt that hunting had grown more difficult, versus 12.0% who thought it was similar, and 17.8% who felt it had improved; 66.2% felt that extraction of wood had grown more difficult versus 11.8% similar and 19.0% improved; and 48.2% felt access to forest products had diminished versus 19.5% who felt it was similar and 32.3% who thought things had improved. Multiple KIs reported that these were still common activities for community members but the practices had changed over the past five years. One education professional explained, “*Yes, they continue hunting... before it was to eat, but now it’s for business… before they killed for the family to eat, now they kill a picuro [large rodent], they sell it and buy tuna to eat at home*” (0103). He continued to describe the effect this had on the availability of food, saying that, “*There are no animals in the forest. Before we hunted to eat, our market was the forest. We didn’t need to go to Puerto Maldonado to buy food or medicines because we had everything here, but now no, this has all been destroyed*” (0103).

Descriptions of poor water quality included “*contamination*”, “*yellow water*”, “*sediment in water*”, and reports that some communities need to boil water or use gorge or rain water for consumption. In some communities, key informants reported that the poor quality and management of the water tank, along with the lack of a sewage infrastructure, regularly caused diarrhea. One health worker who served as a KI in the project described this, “*Diarrhea [is common] because there is no sewage, we don’t have potable water, we don’t have basic services*” (0401). Another KI reported that their community had a sewage infrastructure but that this was not available to all residents. There was a general opinion that residents were unconcerned with the degradation of their environment. One KI commented that “*we are indifferent to our environment, no?* W*e do not see what is happening with our environment*” (0103). However, community members also reported increases in foods not previously available in their communities including the “*vegetables coming fresh*” (0202) such as fruits and vegetables resulting from increased market access and improved transport. One KI described a benefit of the IOH as “*especially in the food also, before you only ate papaya, you ate banana, yucca, now you eat chicken, meat, they bring sausages*” (0401). Overall respondents from KIIs and FGs perceived that the IOH had had a positive impact on nutrition.

## 4. Discussion

Our results suggest that people in the communities surround the IOH in Madre de Dios, Peru link subjective well-being not only to health and income, but also to their community and environment. We found a range of identified impacts of the IOH on well-being. These included positive impacts such as increased access to infrastructure and employment opportunities; and easier access to health services and negative impacts such as a reduction of natural resources for fishing, hunting, resource extraction; and increased migration. Overall, however, community members felt that the perceived negative environmental and social impacts of the IOH were mitigated or maybe even outweighed by benefits related to employment, income, and greater access and integration. They therefore believed that their families’ lives had been improved by the IOH and were generally optimistic about the future. Participants identified the ability to learn new skills and obtain a better education for their children as key to increasing future SWB, emphasizing the proactive and optimistic perspective of this population.

The emphasis on health as a major perceived determinant of SWB is consistent with global literature [35,36,37,38]. As has also been reported in high-income countries [39], new road construction was perceived as having mixed health benefits and drawbacks. Road injury is an increasingly important cause of years of life lost both in Peru, and throughout low- and middle-income countries [40]. Injuries resulting from road accidents, violence, drug and alcohol abuse and other rapid changes in the health care needs in these communities have the potential to add pressure to already over-worked systems with regards to coverage, expertise, and infrastructure [34]. There is a need to support local health systems to deal with this changing burden [41,42]. Improved continuity of care and training of healthcare providers in local health posts could improve confidence in the healthcare system and to accommodate the diversity of patient groups and needs. Increased mentor and peer support of the SERUM programme and other healthcare providers could help further improve service, as well as giving SERUM physicians the chance of applying for work in the post where they have worked in the civil service [34].

Food security was strongly associated with SWB in survey results, and the inclusion of food security in the multivariable regression attenuated the relationship between wealth and SWB. This is generally consistent with the theory that the relationship between monetary income and well-being can be explained in terms of the capacity to meet basic needs [8]. Although the questions used to assess food security in the survey were developed based on FGDs and were correspondingly reflective of local experience, further research into food security and food sovereignty using validated food security measures is needed. The research team also did not prompt participants to describe how their dietary habits had changed because of the IOH. Prior studies have found that the increased market integration associated with road access may be associated with both positive and negative impacts on nutritional status [43]. Therefore health campaigns to target nutritional education around the introduction of ‘new’ food groups and systems in the context of protecting and promoting existing food sovereignty may be important for this context [44]. In this population health campaigns were well-received, especially when organised by the local government.

Our results regarding the determinants of SWB are also generally consistent with the economics of happiness literature, which reports that absolute income is particularly critical to SWB among low income households [8]. Participants generally felt that their economic opportunities were increasing, a trend that is representative of general progress in Peru, although a remaining third of the Peruvian population still lives below the poverty line [34,45]. An area that warrants further research is around financing and options for sustainable lending, as participants felt that the lack of access to credit, income growth, and land titling was a barrier for improving their well-being. This is also consistent with the ‘Allin Kawsay’ concept and other research suggesting that land is necessary for providing livelihoods, dietary diversity and security, which in turn is necessary for fulfilling local conceptions of well-being [11,46].

We found no significant differences in SWB scores by community, gender or age. However, one of the largest changes associated with the IOH was the large influx of migrants to the communities. Although we failed to find evidence that SWB was lower among migrants, there were differences in the determinants of SWB between migrants and non-migrants. Migrants reported lower levels of social capital, by measures of community group participation, trust in local leadership, and sense that their voice mattered. Negative perceptions of migrants by healthcare providers were also reported, which may decrease access to health for this group. Given that the migrants were considered to have lower levels of social capital, interventions that promote the integration of migrants into existing communities and strengthen local institutions may be critical so that communities are empowered to deal with the threats to their well-being brought about by a large influx of new residents [19].

Study limitations include sampling during qualitative data collection, as participants who managed their households were over represented due to availability during scheduled FGDs, thus limiting the diversity of participants. The recruitment and sampling of the KIs will have also contributed to the under-representation of migrants in the qualitative portion of the study since migrants are less represented in community leadership/authority positions. Within the qualitative portion there was a bias towards a negative view of migrants. Due to the under-representation of migrants in the qualitative portion of the study, SWB was not explored from the migrant perspective. Although our survey sampling was exhaustive in that all occupied households in each community were invited to participate, it was also cross-sectional. As a result, we are unable to examine trends in SWB over time and, instead, we report only on changes as perceived by respondents at a single time point. Although there was also a high response rate for the survey there were a large number of homes that were unoccupied at the time of the survey, likely representing highly mobile households who were not present in the community during data collection [19]. An additional limitation is that we did not explore the effect of gold mining on SWB within this study, at the request of community leaders. However, several communities were proximate to gold mines, which are associated with decreased availability of hunting, fishing and consumable forest products. Recent studies have also shown dramatic increases in dietary exposure to mercury, due to mining, in this region [18]. Mercury is a neurotoxin that produces adverse neurological impacts in adults and is particularly detrimental to infant and child development [47]. Finally, our definition of SWB was limited to life evaluation. Further studies should include other SWB other measures or approaches, including emotional wellbeing and eudaimonic wellbeing.

Our integrated, mixed-methods study provided distinct, and complementary, qualitative and quantitative data. Both qualitative and quantitative data reinforced the importance of health and income as locally-meaningful determinates of SWB, but several factors prominent in the qualitative data collection, including land ownership, and access to loans, were less frequently associated with reported SWB in survey results. On the other hand, household food security was a strong determinant of SWB in survey data, but it was infrequently referenced in FGDs and KIIs, suggesting that the directly perceived impact of this factor was low. The relatively low significance of access to loans and land ownership in the survey may relate to the fact that participants may have felt that ‘more money’ better represented what would help them move up the Happy Ladder, whereas the FGDs and interviews gave participants an opportunity to describe what ‘more money’ means within this context. More generally, these qualitatively expressed concerns over financial capital, such as the need for formal land titling and increased access to loans or credit, are reflective of community responses to rapid market integration. The related but different data collected from qualitative versus quantitative tools suggest that the methodologies employed in this project are complementary and contribute to the wider discussion on using mixed methods to inform discussions around SWB [46,48].

## 5. Conclusions

This exploratory study allows us to operationalize the evolving construct of well-being in our study site. Our results suggest that interventions to promote well-being in this region should include locally led health campaigns focused on nutrition and food sovereignty, activities to improve basic infrastructure and support for local health systems to adapt to the rapidly changing environment through improved continuity of care, and mentoring and training opportunities. Based on our results, further research should explore options for financing and sustainable lending in these communities. This research can be used when making recommendations for new highways and infrastructures in similar communities as well-being should be a consideration for both new and existing populations.

## Figures and Tables

**Table 1:** Qualitative data collection summary.

Community	Total Focus Group Discussion Participants	# of Females in FGDs	Key Informant Interviewees	# of Female KIs
La Novia ^a^	12	6	7	4
Alegria ^a^	12	8	6	1
Florida Baja ^a^	15	7	4	2
Santa Rosa ^a^	10	6	5	1
Planchon	20	13	4	0
Mavila	4	0	4	2
Santa Rita Baja	7	7	2	2
Union Progreso	3	3	2	2
Total	73	50	34	14

^a^ These were the four communities selected for the larger trial and where we continued to collect qualitative and quantitative data after 2014. La Novia and Alegría are north-east of Puerto Maldonado, en route to Bolivia; Florida Baja and Santa Rosa are south-west, en route to Cusco.

**Table 2:** Socio-demographic characteristics of survey respondents.

	La Novia	Alegria	Florida Baja	Santa Rosa	Overall
Total HHs	64	263	25	170	522
Total Individuals	264	1040	104	687	2077
Respondent sex (percent male)	32.8%	28.9%	28.0%	27.7%	71.1%
Respondent age (mean SD)	38.1 (11.9)	36.6 (13.3)	34.1 (12.1)	35.5 (12.6)	36.0 (12.9)
Head of household migration history (% migrant)	57.8%	53.2%	76.0%	72.9%	61.3%
Happy Ladder (SWB) (median, IQR)	4 (2, 5)	4 (3, 6)	5 (3, 5)	4 (3, 5)	4 (3, 5)
Expected Happy Ladder (SWB) in four years (median, IQR)	6 (4, 7)	6 (4, 8)	6 (4, 8)	6 (4, 8)	6 (4, 8)

**Table 3:** Multivariate Regression Results: Specific Factors as per survey (derived from initial FGD & theory-driven).

Form of Capital	Factors	Variable	Prevalence	Bivariate Linear Regression Coefficient	Multiple Linear Regression Coefficient
Social	Participation in community groups	Any individual in household participates in a community group	18.4%	0.66 (0.20, 1.12)(*p* = 0.005)	0.51 (0.06, 0.97)(*p* = 0.027)
	Perception of corruption	Perceives local government to be less corrupt	38.7%	0.38 (0.01, 0.75)(*p* = 0.045)	0.30 (−0.06, 0.66)(*p* = 0.098)
		Perceives regional government to be less corrupt	18.6%	0.08 (−0.39, 0.54)(*p* = 0.743)	-
		Perceives national government to be less corrupt	14.6%	0.05 (−0.47, 0.56)(*p* = 0.861)	-
	Migration status	Community member who self-identified as living in the community after the construction of the IOH (equivalent to having moved to the community within the past 10 years)	61.3%	−0.18 (−0.55, 0.19)(*p* = 0.349)	-
	Decision-making power	Thinks that their opinion is valued in the community	55.2%	0.45 (0.09, 0.81)(*p* = 0.015)	-
		Feels that community has influence in decisions about land use	24.0%	0.30 (−0.15, 0.70)(*p* = 0.198)	-
Human	Age	Average of head of household	39.4 (12.7)	−0.01 (−0.02, 0.01)(*p* = 0.297)	-
	Gender	Male head of household	88.7%	−0.28 (−0.56, 0.01)(*p* = 0.057)	0.26 (−0.01, 0.53)(*p* = 0.062)
	Marital Status	Head of household is married	79.1%	0.59 (0.15, 1.03)(*p* = 0.009)	-
	Education	Head of household has some secondary education or more	63.4%	0.27 (-0.11, 0.64)(*p* = 0.159)	-
	Household size	Number of individuals in household	4.0 (3, 5)	0.18 (0.07, 0.28)(*p* = 0.001)	0.16 (0.06, 0.26)(*p* = 0.002)
	Healthy in past year	No health issues reported	66.5%	−0.29 (−0.68, 0.09)(*p* = 0.130)	-
	Health insurance	Household is enrolled in ‘SIS’	68.8%	−0.14 (−0.53, 0.25)(*p* = 0.491)	-
	Time get to get to health care	Able to reach first point of care in <=1 h (only among those HHs that faced a health problem)	55.9%	Na	-
Financial	Wealth	Wealth Index	0.4 (0.15)	1.87 (0.64, 3.10)(*p* = 0.003)	1.02 (−0.30, 2.34)(*p* = 0.130)
	Home ownership	Owns home	68.0%	−0.03 (−0.42, 0.36)(*p* = 0.872)	-
	Access to land	Owns/rents >1 hectare of land	31.2%	0.32 (−0.06, 0.71)(*p* = 0.102)	-
Physical	Natural resources	Enters woods regularly	54.8%	0.37 (0.01, 0.73)(*p* = 0.046)	-
	Air and water quality	Considers water to be clean	28.5%	0.24 (−0.16, 0.64)(*p* = 0.245)	-
		Considered air to be clean	19.4%	0.35 (−0.11, 0.80)(*p* = 0.133)	-
	Food security	Fully food secure	29.3%	0.78 (0.39, 1.17)(*p* < 0.001)	0.72 (0.33, 1.10)(*p* < 0.001)
	Water security	Has access to improved drinking water	84.5%	0.67 (0.18, 1.16)(*p* = 0.007)	-
Personal	Security	Feels secure in community	63.0%	0.49 (0.11, 0.86)(*p* = 0.010)	0.42 (0.06, 0.78)(*p* = 0.023)
